# Supplementary material for: Destructiveness of pyroclastic surges controlled by turbulent fluctuations
Source: Nat Commun. 2021 Dec 15;12:7306. doi: 10.1038/s41467-021-27517-9 (PMC8674289; doi:10.1038/s41467-021-27517-9)
Supplement: Supplementary file 1 — Supplementary Information [file 41467_2021_27517_MOESM1_ESM.pdf]

Supplementary Material

Destructiveness of pyroclastic surges controlled by turbulent fluctuations

Ermanno Brosch[1]*, Gert Lube[1], Matteo Cerminara[2], Tomaso Esposti-Ongaro[2], Eric C.P. Breard[3], Josef Dufek[3], Betty Sovilla[4] and Luke Fullard[5]

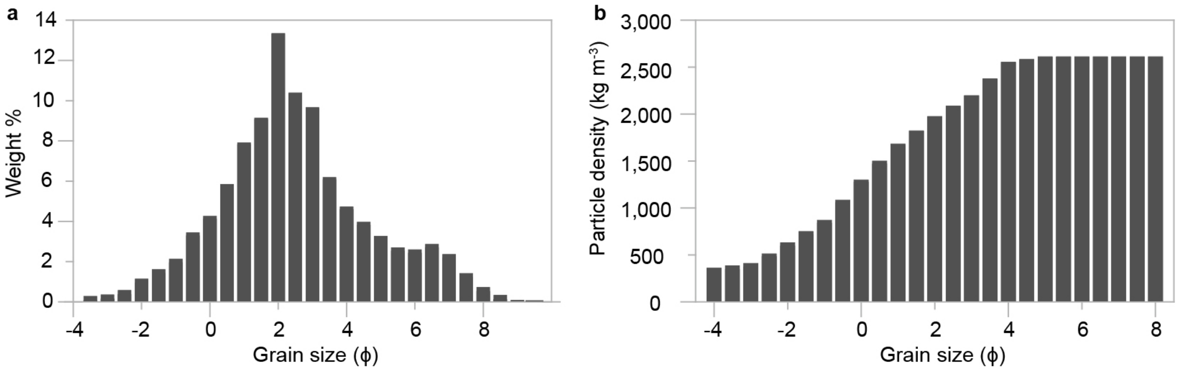

Supplementary Fig. 1 | Grain size and particle density distributions of the initial mixture. a, grain size distribution of the initial experimental mixture. **b**, density distribution of the natural volcanic particles of the Taupo ignimbrite[1] composing the experimental mixture.

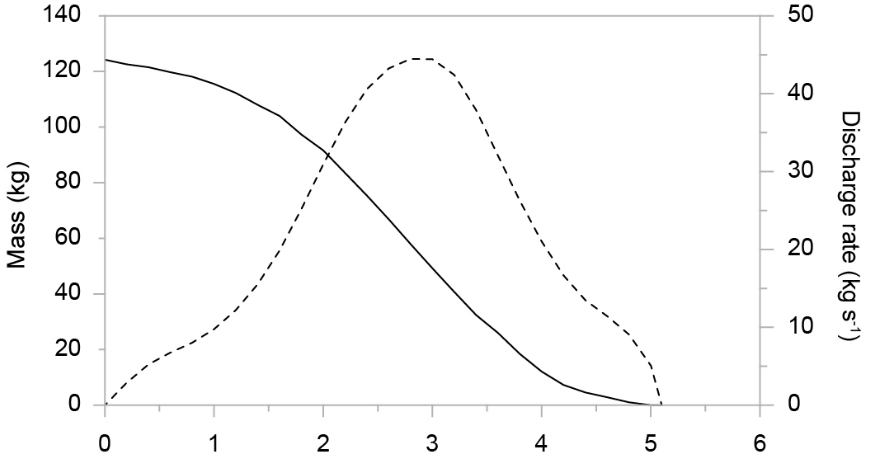

Supplementary Fig. 2 | Mixture discharge and discharge rate. The hopper discharge, initiating the experiment (solid line), lasts c. 5.2 seconds. The resulting mass discharge rate (dashed line) is approximately unimodal with a time-average value of c. 24 kg s^{-1} and a maximum value of c. 44 kg s^{-1}.

Supplementary Table 1 | Experimental conditions. The initial and boundary conditions of the large-scale experiment investigated in this study.

Parameter	Experimental condition
Initial mass	124 kg
Grain size distribution	0.002–16 mm
Fine-ash content (<0.063 mm)	20 wt.%
Mixture temperature	120 °C
Substrate roughness	0.005 m
Drop height	7 m
Impact velocity	c. 7 ms^{-1}
Channel width	0.5 m
Channel inclination	6°

Supplementary Table 2 | Ranges in parameters and non-dimensional products for Figure 9. The table lists the average flow height L_{ave} and average flow velocity U_{ave}, the ranges in flow height L_{range} and flow velocity U_{range}, characteristic large-eddy frequencies f, computed Strouhal numbers Str, and bulk flow Reynolds numbers Re of the analysed (natural and experimental) PDCs and powder snow avalanche.

Flow	Ref.	L_{ave} (m)	U_{ave} (ms^{-1})	L_{range} (m)	U_{range} (ms^{-1})	f (Hz)	Str	Re
PELE pyroclastic surges	This study	1.2	4.8	0.95–1.5	4.12–6.52	1.249	0.312	1.5×10^6
Mount St. Helens, 1980	Ref.[2]	750	93	500–1000	72–104	0.035	0.282	1.2×10^9
Whakaari / White Island, 2019	This study	26	17	18–28	12–19	0.199	0.305	8.7×10^6
Powder snow avalanche #20163017, 2016	Ref.[3,4]	20	40	14–25	31–47	0.6	0.3	10^6–10^9
Te Maari, Tongariro, 2012	Ref.[5]			60–80	10–20			2.3×10^7
Montserrat, Boxing Day, 1997	Ref.[6]			100–200	40–60			5.1×10^8

Supplementary Note 1 | Dimensional scaling of the velocity of density discontinuities and of the ratio of maximum dynamic pressures to mean dynamic pressures in pyroclastic surges

From dimensional arguments, frequency must scale with the ratio of the characteristic velocity scale to the characteristic length scale of the gravity current. Thus, the two frequencies f_{max} = 1.75 Hz and f = 1.25 Hz associated with the largest dynamic pressures at the top of the turbulent energy cascade can be expressed as:

$$f_{max} \sim \frac{c}{L_{ave}} \quad (1)$$

$$f \sim \frac{U_{ave}}{L_{ave}} \quad (2)$$

Substitution of Eq. 1 and Eq. 2 yields

$$\frac{f_{max}}{f} = \frac{c}{U_{ave}} \quad (3)$$

which is Eq. 8 in the main text, and from which c can be computed to demonstrate that it corresponds with the experimentally measured average velocity of the density continuities.

Density discontinuities in shallow flows travel at the velocity of gravity waves. The velocities c and U_{ave} can be expressed as

$$c \sim \sqrt{\rho_{max} L_{ave}} \quad (4)$$

$$U_{ave} \sim \sqrt{\rho_c L_{ave}} \quad (5)$$

where ρ_{max} is the maximum flow density associated with the density discontinuities. Substitution of Eq. 4 and Eq. 5 yields

$$\frac{\rho_{max}}{\rho_C} \sim \left(\frac{c}{U_{ave}}\right)^2 \text{ and thus} \quad (6)$$

$$\frac{P_{max}}{P_{ave}} = \frac{\rho_{max} c^2}{\rho_{ave} U_{ave}^2} \sim \left(\frac{c}{U_{ave}}\right)^4 \quad (7)$$

which is Eq. 9 in the main text, and whose solution, through experimentally measured values of c an U_{ave}, corresponds with the experimentally measured ratio P_{max}/P_{ave} in the wall region of the experimental pyroclastic surge.

Supplementary References

1. Wilson CJN. The Taupo Eruption, New Zealand. II. The Taupo Ignimbrite. *Philosophical Transactions of the Royal Society of London Series A, Mathematical and Physical Sciences* 314, 229-310 (1985).

2. Esposti Ongaro T, Clarke AB, Voight B, Neri A, Widiwijayanti C. Multiphase flow dynamics of pyroclastic density currents during the May 18, 1980 lateral blast of Mount St. Helens. *Journal of Geophysical Research: Solid Earth* 117, B06208 (2012).

3. Sovilla B, McElwaine JN, Köhler A. The Intermittency Regions of Powder Snow Avalanches. *Journal of Geophysical Research: Earth Surface* 123, 2525–2545 (2018).

4. Sovilla B, McElwaine JN, Köhler A. The Intermittency Regions of Powder Snow Avalanches [Data-set]. Zenodo (2018).

5. Breard ECP, Lube G, Cronin SJ, Valentine GA. Transport and deposition processes of the hydrothermal blast of the 6 August 2012 Te Maari eruption, Mt. Tongariro. *Bulletin of Volcanology* 77, 1-18 (2015).

6. Esposti Ongaro T, Clarke AB, Neri A, Voight B, Widiwijayanti C. Fluid dynamics of the 1997 Boxing Day volcanic blast on Montserrat, West Indies. *Journal of Geophysical Research* 113, B03211 (2008).
