# Supplementary material for: Destructiveness of pyroclastic surges controlled by turbulent fluctuations
Source: Nat Commun. 2021 Dec 15;12:7306. doi: 10.1038/s41467-021-27517-9 (PMC8674289; doi:10.1038/s41467-021-27517-9)
Supplement: Supplementary file 3 — Description of Additional Supplementary Files [file 41467_2021_27517_MOESM3_ESM.pdf]

Description of Additional Supplementary Files

File name: Supplementary Movie 1.mp4

Description: Overview of the generated experimental pyroclastic surge.

Sequence 1: View of the advancing current inside the channel confined part of the runout section.

Sequence 2: View of the current transitioning from the channel confined to the unconfined runout including the late-stage buoyant rise of the hot mixture.

Sequence 3: High speed video sequence showing the passage of the lower part of the turbulent current and the basal bedload region at a static observer location of 5.77 m.

File name: Supplementary Movie 2.mp4

Description: Time-variant vertical velocity profiles at the static observer location 3.12 m.

This composite movie depicts, on the left-hand side, a high-speed camera sequence showing the passage of the experimental pyroclastic density current above the height-variant bedload layer (separated by the red line) and the basal aggrading deposit (separated by the blue line).

Synchronised with this footage, and shown on the right-hand side, are the time-variant vertical velocity profiles (black lines) at this location (white line on left hand side) and the time-variant mean vertical velocity profiles (Eq. 2, green line).
